# The Effects and Differences of Social Support, Depression, and Vital Exhaustion during the COVID-19 Pandemic among International and Domestic University Students

**DOI:** 10.3390/ijerph20021502

**Published:** 2023-01-13

**Authors:** Viktor Rekenyi, Szabolcs Garbóczy, Anita Szemán-Nagy, Ala’a B. Al-Tammemi, Mohamed Sayed-Ahmad, László Robert Kolozsvári

**Affiliations:** 1 Doctoral School of Health Sciences, University of Debrecen, 4032 Debrecen, Hungary; 2 Department of Family and Occupational Medicine, Faculty of Medicine, University of Debrecen, 4032 Debrecen, Hungary; 3 Department of Psychiatry, Faculty of Medicine, University of Debrecen, 4032 Debrecen, Hungary; 4 Department of Personality and Clinical Psychology, Institute of Psychology, University of Debrecen, 4032 Debrecen, Hungary; 5 Applied Science Research Center, Applied Science Private University, Amman 11931, Jordan; 6 Faculty of Medicine, University of Debrecen, 4032 Debrecen, Hungary

**Keywords:** international students, domestic students, social support, depression, vital exhaustion

## Abstract

Background: Our study aimed to assess the differences between domestic and international students in terms of social support, vital exhaustion, and depression during the period of COVID-19 and to examine the relationships and potential effects of these factors on each other. Methods: The online cross-sectional survey was conducted via Google Forms^®^ at three time intervals during the pandemic. Results: Here, 1320, 246, and 139 students completed our questionnaires in the different time intervals. The international students reported significantly lower values in terms of perceived social support. Concerning depression, the international female students reported higher values than the domestic female students. Significant correlations were found in both samples between vital exhaustion and depression, as well as between perceived social support and depression. Conclusion: In this study, the international students reported lower levels of perceived social support and higher levels of depression, particularly among females. The correlations between depression, social support, and vital exhaustion might highlight protective and risk factors. These findings emphasize the importance of addressing social support and mental health among university students, especially among international students who have a difficult time finding social support during times of stress, such as during the COVID-19 pandemic.

## 1. Background

The first cases of coronavirus disease were declared on 4 March 2020 in Hungary, and shortly after the educational institutes switched their teaching method to the online form and a nationwide lockdown was announced [1].

### Theoretical and Conceptual Framework

Anti-pandemic regulations can disrupt social interconnectedness, which is associated with an increased risk of psychological difficulties [2]. During these times, one particular topic has been the focus of interest, which was the role of social support as a psychosocial protective factor. Several studies have addressed the relationship between mental health and social support and found that the presence of social support can be considered a protective factor that predicts better mental health functioning and helps prevent the development of mental health issues [3,4].

Previous researchers have found that college students are more likely to have mental health problems [5] and also that the prevalence of depressive symptoms among them is higher than among the general population or non-college students [6]. College students with depressive symptoms are less likely to participate in organized activities compared with healthy ones, which may negatively impact their status in social networks and even increase their suicidal tendencies [7]. Social support has been described to have a protective effect on college student’s mental health, and its inadequacy may also increase the risk of depressive symptoms [8,9].

The fact that international students who study in a host country away from home have more mental health problems than those who study in their home country has long been supported by research, which has already drawn attention to the importance of social support, which can alleviate mental health problems for international students as well [10,11,12].

Concerning depression, men have a lower prevalence rate than women but a higher suicide rate, and because of this many kinds of research investigate gender differences regarding depression to be able to adequately detect it [13,14].

The conceptual separation of depression and vital exhaustion has been debated in the literature for years [15,16]. In any case, we went beyond these debates and together examined vital exhaustion and depression. Vital exhaustion is a mental state characterized by extreme fatigue, increased irritability, and feelings of demoralization. It is considered to be a potential response to intractable problems in individuals’ lives, particularly when they are not able to adapt to prolonged exposure to psychological stressors [17].

The purpose of our study was to compare the levels of perceived social support, vital exhaustion, and depression and to examine their relationships to each other among university students in Hungary during the COVID-19 pandemic in three periods, using three validated assessment tools for each factor. By conducting the survey in three different time periods, we were able to observe the differences and relationships among the examined factors caused by the effects of changing circumstances related to the pandemic within the rounds. We wanted to see the differences not only in terms of the student status (domestic vs. international) but also in terms of the gender. We hypothesized that students away from home receive less social support, and this may be related to the degree of depression and vital exhaustion. Furthermore, we believed that it is easier for women to find social support that would be thought to counteract depression [18]. Because there is more evidence that women have a higher incidence rate of depression, we tried to refute this discrepancy by using a more specific questionnaire among men, and we expected that there would be no significant gender difference in the severity of depression [19,20]. We believe that the presence of social support can act as a protective factor against the onset of mental health difficulties, and that examining these relationships can provide insights into the potential impacts of social support on mental health outcomes during the COVID-19 pandemic. We also hypothesized that higher levels of vital exhaustion would be positively correlated with higher levels of depression.

## 2. Methods

### 2.1. Data Collection

Our study used a cross-sectional design in three time intervals with the help of self-administered questionnaires we created in Google Forms.

The first round of the survey administration process was carried out in the period between 30 April 2020 and 15 May 2020. At the start of the pandemic, it was often claimed that the virus was brought into the country by foreigners or Hungarians traveling home from abroad [21].

The second round took place between 15 June 2020 and 29 June 2020. During this phase, there were perhaps the fewest new cases, the lowest rate of positive tests, and minimal deaths. The restrictions had been lifted almost without exception [21].

The third round of questionnaires was filled out by the participants during the period between 20 July 2021 and 7 August 2021. During this time, the country was past two very big waves, and the third wave’s downturn took place. Vaccinations had been released, which were also available to international students, and the Head of the Prime Minister’s Office ranked Hungary as one of the safest countries [21].

### 2.2. Participants

We conducted our study at the second largest university in Hungary. According to the university’s website, there are 28,593 students, of which at least 6297 are international students [22].

International and Hungarian students were included in our sampling. The targets were reached through social media platforms (e.g., Facebook), and also the university’s official student administration system (Neptun) was used to reach out to them. We assumed that our survey reached every student at the university. The questionnaires could be filled out anonymously during the determined periods, meaning of a convenient sampling approach was utilized.

### 2.3. Measures

Four international scales were applied in the development of the survey, collecting data about perceived social support, vital exhaustion, and male and female depression. Domestic students filled out Hungarian versions of the questionnaires, while international students were asked to complete English versions of them.

To assess the amount of social support, we used the *Multidimensional Scale of Perceived Social Support (MSPSS)*. The 12 items are rated on a seven-point Likert scale in the English version, while the 10-item Hungarian version uses a five-point Likert scale. Three subscales were identified, each addressing a different source of support: family, friends, and significant others [23]. The original version of the questionnaire has high internal consistency both in English (Cronbach-α = 0.88) and Hungarian (Cronbach-α = 0.91) versions [23,24].

The Shortened Maastricht Vital Exhaustion Questionnaire and its Hungarian version served as tools to assess vital exhaustion. Vital exhaustion is related to an individual’s general well-being, which is measured using five dichotomic items. The internal consistency of the item group proved to be good for the Hungarian validation (Cronbach- α =0.78) [25], as well as for the English version of the questionnaire (Cronbach- α = 0.86) [26].

Female depressive symptoms in the sample were assessed using the revised version of the 21-item Beck Depression Inventory (BDI). The participants are asked to rate the range to which they have experienced particular depressive symptoms in the past week using a four-point Likert scale [27,28].

The level of male depression was measured with the Gotland Male Depression Scale (GMDS). Both the English and Hungarian versions of the questionnaire have 13 items, and it assesses not only the so-called “traditional” depressive symptoms but male depressive symptoms as well (lower stress threshold, aggression, substance abuse, over-involvement in work or sports). The respondents used a four-point Likert scale to answer the questions [29,30].

To compare the extent of depression between the genders, we divided the results into three groups according to previous recommendations [27,31]: no, minimal-mild, or moderate-severe depression. The recommended point limits for the BDI are respectively 0–13, 14–28, and 29–63; and for the GMDS are respectively 0–13, 14–26, and 27–39.

### 2.4. Analysis

The required data were extracted from Google Forms in an Excel sheet. First, we checked the quality of the data and coded it in a format that we could analyze with SPSS (v.25). The presentation of the descriptive and summary statistics was appropriate. To appraise the differences between groups (nationality, gender) in accordance with the level of perceived social support, vital exhaustion, and depression, we used non-parametric Mann–Whitney U tests, since the variables had no normal distributions. We also used Spearman’s rank correlation to evaluate the relationship between the investigated factors within the international and Hungarian groups. A *p*-value of less than 0.05 was set for statistical significance.

### 2.5. Ethical Considerations

The Hungarian Ethical Review Committee for Research in Psychology provided the ethical permission (Reference number: 2020-45).

## 3. Results

The demographic characteristics are shown in Table 1.

### 3.1. Perceived Social Support

The perceived social support levels of the university students were investigated according to gender via Mann–Whitney U test in each round and also in both samples.

**Figure 1:**
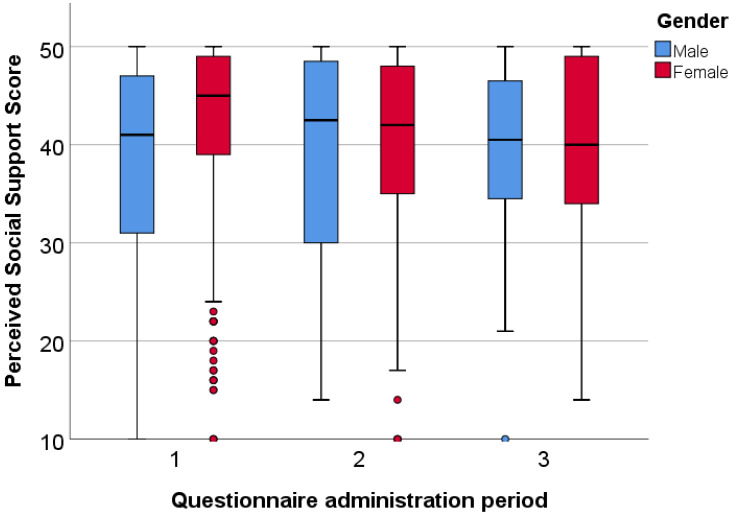
Gender differences in perceived social support among Hungarian students.

The examination of Hungarian students’ perceived social support is shown in Figure 1, which shows that the Hungarian female students had significantly higher values (M = 45) than male students (M = 41) in the first round of our survey (*p* < 0.001). The other rounds showed no significant differences between genders.

We also investigated gender differences among Hungarian students according to the MSPSS subscales. The perceived social support subscales differed significantly based on gender, as women had significantly higher values in the first round in all aspects of the questionnaire (Table 2).

**Figure 2:**
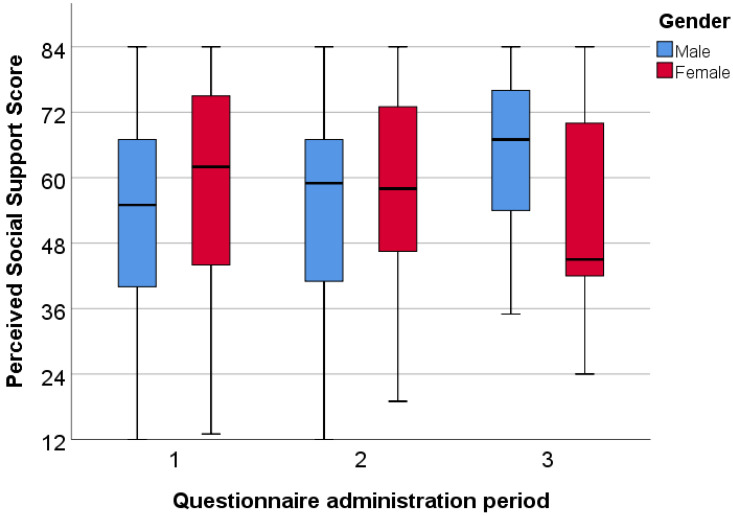
Gender differences in perceived social support among international students.

In the international sample, regarding the first round of questionnaire administration, the female students had a significantly (*p* = 0.05) higher median (62) than the male students (55). In the last period, the median for the female students was 45 compared to 67 for the male students (*p* = 0.036) (Figure 2).

Similar to the Hungarian sample, the international women’s perceived social support subscale levels were higher in the first round than the men’s, although only significant the family and significant others subscales were significant. We also found significantly higher values in the female group during the second period of administration in the friends subscale. In contrast to these findings, the values for men’s perceived social support from their family and friends were significantly higher in the third round (Table 3).

As the Hungarian and English versions of the MSPSS differed both in the numbers of items and scales, we converted the results into percentages, which allowed us to compare the samples.

**Figure 3:**
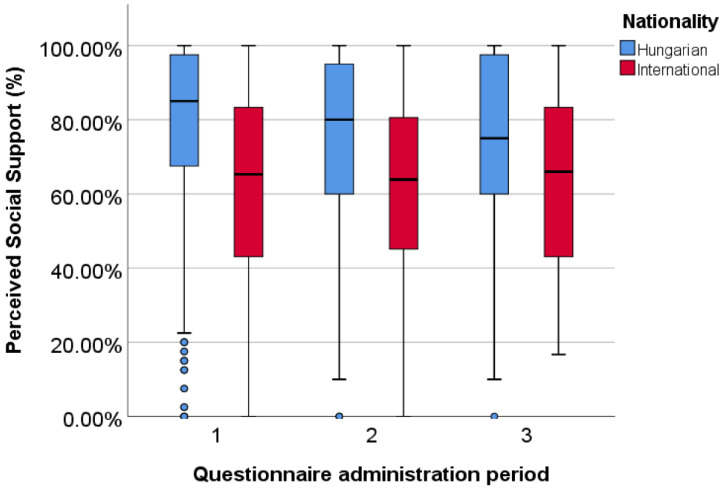
Differences in perceived social support among domestic and international samples.

In the first period of administration, the Hungarian students reached an 85.00% median compared to the international student’s 65.28% (*p* < 0.001). During the second round, the Hungarian level of perceived social support was also higher (M = 80.00%) than the international students’ (M = 63.89%) (*p* < 0,001). Similar to the first two rounds in the last period, domestic students also reached higher scores (M = 75.00%) than international students (65.97%), although the difference was not significant (*p* = 0.056) (Figure 3).

Comparing the two samples in terms of the subscales, the international students had lower values for social support in all of the examined factors. The results were significant in every subscale during the first period of questionnaire administration, as well as in the second and third rounds for the friends and significant others subscales (Table 4).

### 3.2. Vital Exhaustion

**Figure 4:**
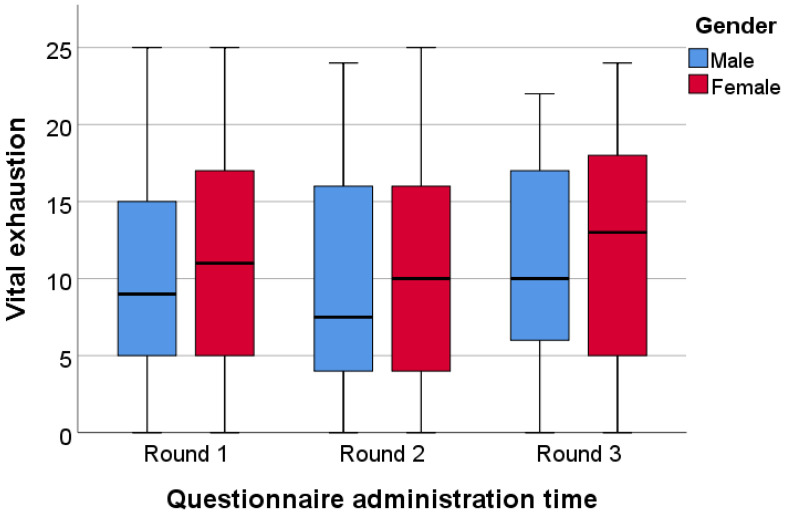
Gender differences in vital exhaustion among Hungarian students.

Examining the level of vital exhaustion regarding genders in the Hungarian sample, we found that women had higher values in each round (Figure 4), although this result was only significant in the first period of administration. In this case, the median for female students was 11.00, while the male students’ median was 9.00 (*p* < 0.001).

**Figure 5:**
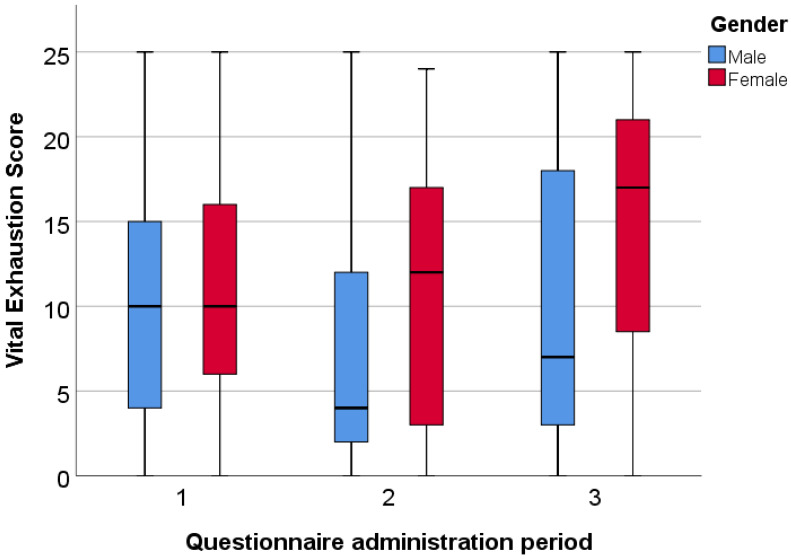
Gender differences in vital exhaustion among international students.

In the international sample, similar results can be observed (Figure 5). Women reached higher average scores in every round, and the difference was significant in the second period, with a median of 12.00 compared to the male students with 4.00 (*p* = 0.022).

There was no significant difference between the Hungarian and international students concerning vital exhaustion.

### 3.3. Depression

According to the results of the BDI completed by the females, the average scores for international students were significantly higher than for the domestic students in every period. There were no significant differences in GMDS scores between the two samples. The results of the depression questionnaires are shown in Table 5.

As mentioned previously, both scales can be aggregated into 3 groups according to the scores, allowing us to examine the differences between the genders in terms of depression. In the Hungarian group, there were no significant differences and both genders’ medians were the same. Among the international students, the women suffered from significantly more severe depressive symptoms in the first round; this difference decreased during the second round, while the difference was not significant in the third round (Table 6).

### 3.4. Correlations

We correlated the vital exhaustion, perceived social support, and depression values using Spearman’s rank correlation (Table 7). Significant positive correlations can be observed in both samples between vital exhaustion and depression, and moderate negative correlations were associated with perceived social support and depression.

## 4. Discussion

Previous studies have proven that social networks among individuals play an essential role in mitigating psychological problems [32,33]. As expected, we found significant differences in perceived social support among domestic and international students at the University of Debrecen. According to our findings, international students felt that they acquire less social support from family, friends, and also special persons in their lives. The international students’ lack of perceived social support compared to the domestic students was in line with previous investigations [34,35].

Based on the previous investigations [36,37], our results indicate that female and male university students tend to differ in their perceptions of support availability, with female students obtaining higher total scores in the first period of the questionnaire administration process. The indications from some studies show that females do receive more support than males and they are more likely to demand and also provide support [38,39].

International students are prone to being exposed to more psychological stressors because of the separation from their home environment and their different cultural values, language, levels of academic preparation, and study habits; they also tend to have less social support [40,41], which can be expected to lead to higher levels of vital exhaustion, although this study could not confirm a difference in vital exhaustion levels among domestic and international students. In our present study, we found significantly higher vital exhaustion levels among females in the first period of survey administration in the Hungarian sample, and also in the international sample during the second round.

Among university students, depression is one of the most prevalent psychological disorders [42,43]. International students suffer more from depressive symptoms, and it can be the consequence of multiple sources such as a lack of social support, the adverse effects of high vital exhaustion values, and also isolation and loneliness [34,35,44,45]. Our findings also suggest that depressive symptoms are more prevalent among international students, although this was only significant for female students. Moreover, in the gender comparison, we found a significant difference only between international students, and women suffered more from depressive symptoms; only in the first and to a lesser extent in the second measurement in the third round were the differences no longer significant.

Strong positive correlations were observed regarding depression and vital exhaustion in both samples, which indicates the potential role of vital exhaustion as a risk factor related to depression. On the other hand, the moderate negative correlations between depression and perceived social support designate one’s perceived social support as a protective factor and a moderator against depression among university students.

### New Contribution to the Literature

The differences between the social support levels of domestic and international students gradually decreased. The reasons behind that phenomenon could be numerous, including the vaccine, which gave the freedom and courage to international students to re-open to the world; the decreasing number of restrictions; and the initial shock caused by the poor quality of media communications having been alleviated.

The shift between the extent of perceived social support and the severity of depressive symptoms is an interesting phenomenon; while international female students perceived greater social support at the onset of the virus, males experienced less, and this difference gradually narrowed. In contrast, the depressive symptoms started higher in women, and the gender gap disappeared again over time. It is worth comparing this phenomenon with the change in correlations between social support and depressive symptoms in international women and men. While initially the correlation was negative for men, it reversed in the third round, and in women there was a huge negative correlation change in the last round.

At the onset of the virus, the domestic students seemed to perceive significantly more social support in all areas than their international counterparts, but social support seemed to level off between domestic and international students as a result of the increasing time spent under the threatening factors. This raises the possibility that in the event of an outbreak of a protracted danger, the provision of well-chosen information and communication and mental support to foreigners by a host country can have particularly constructive effects, and can even serve preventive purposes. This is because social support can counteract vital exhaustion and depression. Regarding depression, the most vulnerable group seemed to be the female international student group. Using different gender-specific questionnaires, it became apparent that after the initial shock, men are just as vulnerable as women when it comes to depression.

Although this study was conducted as a cross-sectional survey and not a longitudinal study, we were able to examine the differences in these factors within the three time periods. Additionally, the sample size was relatively small in the second and third rounds. The results may not be generalizable to the wider population of university students in Hungary. Further research, including with longitudinal studies, is needed to better understand the factors influencing the mental health of university students during the COVID-19 pandemic and after the lifting of restrictions.

## 5. Conclusions

Our cross-sectional study found significant differences in the levels of perceived social support between domestic and international university students in Hungary during the COVID-19 pandemic. The international students reported lower levels of perceived social support in all three periods of the survey. These differences were statistically significant in the first and second rounds, but not in the third round. When examining the subscales of perceived social support, the international students consistently reported lower levels of social support from family, friends, and significant others compared to Hungarian students, with these differences being statistically significant in the first period for all subscales and in the second and third periods for the friends and significant others subscales. These findings suggest that international students may be more vulnerable to experiencing low levels of social support, which could have negative consequences for their mental health as compared to domestic students. There was no significant difference in vital exhaustion levels between Hungarian and international students, although the female students did report significantly higher levels of vital exhaustion in both samples, with this difference being significant in the first round of the survey in the Hungarian sample and in the second round of the survey in the international sample. In terms of depression, the international female students scored higher than Hungarians. The female international students had higher levels of depression than the male international students in every round, but this difference was not statistically significant in the third round. Strong positive correlations were observed between depression and vital exhaustion in both samples, and moderate negative correlations were observed between depression and perceived social support in both samples.

Overall, the results of this study suggest that social support plays a significant role in the mental health of university students during the COVID-19 pandemic. Given the outcomes of this research, it is important to focus on providing mental health resources and support, especially for international students during times of stress and uncertainty. It is crucial to highlight that international students are far away from some of their social support resources and may be more vulnerable to experiencing low levels of social support in a foreign country, which could have negative consequences on their mental health. Our findings also suggest that vital exhaustion may be a risk factor for depression, and that gender may be a contributing factor to the experience of these mental health factors. The strong correlations observed between depression, vital exhaustion, and social support emphasize the importance of addressing these issues in a comprehensive and interconnected manner. The future research should involve a longitudinal study to further examine the dynamic relationships between these factors and the impacts of changing circumstances related to the pandemic.

## Figures and Tables

**Table 1:** Demographic characteristics of the respondents.

Variables	Hungarian	International	Chi-Square Test
1.round	*n* = 948	*n* = 341	
Gender			χ2(1) = 35.06; *p* < 0.001
Female	719 (75.8%)	201 (58.9%)	
Male	229 (24.2%)	140 (41.1%)	
2.round	*n* = 142	*n* = 104	
Gender			χ2(1) = 6.05; *p* = 0.014
Female	102 (71.8%)	59 (56.7%)	
Male	40 (28.2%)	45 (43.3%)	
3.round	*n* = 105	*n* = 34	
Gender			χ2(1) = 5.56; *p* = 0.109
Female	77 (73.3%)	20 (58.8%)	
Male	28 (26.7%)	14 (41.2%)	

**Table 2:** Gender differences in perceived social support subscales among Hungarian students.

Questionnaire Administration Period and Subscale Medians	Male	Female	*p*-Value
Round 1			
Family	15.00	17.00	<0.001
Friends	13.00	14.00	<0.001
Significant others	14.00	15.00	<0.001
Round 2			
Family	16.00	16.50	0.535
Friends	13.50	12.00	0.755
Significant others	15.00	15.00	0.255
Round 3			
Family	16.00	14.00	0.571
Friends	13.50	14.00	0.636
Significant others	14.00	15.00	0.146

**Table 3:** Gender differences in perceived social support subscales among international students.

Questionnaire Administration Period and Subscale Medians	Male	Female	*p*-Value
Round 1			
Family	20.00	22.00	0.047
Friends	19.00	20.00	0.062
Significant others	17.50	21.00	<0.001
Round 2			
Family	21.00	19.00	0.937
Friends	18.00	20.00	0.036
Significant others	19.00	20.00	0.137
Round 3			
Family	22.50	16.50	0.020
Friends	23.50	15.50	0.031
Significant others	25.50	15.50	0.089

**Table 4:** Differences in perceived social support subscales among the domestic and international students.

Questionnaire Administration Period and Subscale Medians	Hungarian	International	*p*-Value
Round 1			
Family	81.25%	70.83%	<0.001
Friends	91.67%	66.67%	<0.001
Significant others	100.00%	66.67%	<0.001
Round 2			
Family	75.00%	66.67%	0.073
Friends	83.33%	66.67%	0.001
Significant others	100.00%	66.67%	<0.001
Round 3			
Family	62.50%	66.67%	0.813
Friends	91.67%	68.75%	0.027
Significant others	100.00%	54.17%	<0.001

**Table 5:** Male and female depression scores.

Questionnaire Administration Period and Subscale Medians	Male			Female		
	Hungarian	International	*p*-Value	Hungarian	International	*p*-Value
Round 1	13.00	13.00	0.347	11.00	17.00	<0.001
Round 2	11.00	10.00	0.217	10.50	15.00	0.018
Round 3	12.50	8.00	0.362	13.00	23.00	0.020

**Table 6:** Differences in depression levels between domestic and international students.

Questionnaire Administration Period and Nationality	Male	Female	*p*-Value
Hungarian			
Round 1	1.00	1.00	0.062
Round 2	1.00	1.00	0.478
Round 3	1.00	1.00	0.707
International			
Round 1	1.00	2.00	0.006
Round 2	1.00	2.00	0.027
Round 3	1.00	2.00	0.259

**Table 7:** Spearman’s rank correlations in the samples.

		Vital Exhaustion	Perceived Social Support
Hungarian			
Round 1	Male depression	0.795 **	−0.219 **
	Female depression	0.763 **	−0.186 **
Round 2	Male depression	0.776 **	−0.088
	Female depression	0.772 **	−0.157
Round 3	Male depression	0.886 **	−0.283 *
	Female depression	0.822 **	−0.058
International			
Round 1	Male depression	0.775 **	−0.208 **
	Female depression	0.702 **	−0.004
Round 2	Male depression	0.693 **	−0.205 *
	Female depression	0.708 **	−0.090
Round 3	Male depression	0.441	0.231
	Female depression	0.509 *	−0.479 *

Note: * *p* < 0.05, ** *p* < 0.001.

## Data Availability

The data presented in this study are available on request from the corresponding author. The data are not publicly available due to ethical reasons.
